# 

**Published:** 1897-12

**Authors:** 


### CONTENTS.

**ORIGINAL COMMUNICATIONS—**

SURGERY OF THE GALL-BLADDER AND DUCTS. By J. McFadden Gaston, M.D., Atlanta, Ga .... 649
THE VALUE OF DRINKING-WATER AS A PHYSIOLOGICAL AND THERAPEUTIC AGENT. By A. J. Kilpatrick, M.D., Augusta, Ga .... 658
COMMON WARTS AND THEIR TREATMENT. By M. B. Hutchins, M.D., Atlanta, Ga .... 663
TENOTOMY IN CONVERGENT SQUINT. By A. Bethune Patterson, M.D., Atlanta, Ga .... 665
THE THERAPEUTIC ADMINISTRATION OF IRON. By L. A. Felder, M.D., Atlanta, Ga .... 667

**SOCIETY REPORTS—**

TRI-STATE MEDICAL SOCIETY OF ALABAMA, GEORGIA AND TENNESSEE—NINTH ANNUAL MEETING .... 669
RICHMOND ACADEMY OF MEDICINE AND SURGERY .... 678

**CORRESPONDENCE—**

EFFICIENT VACCINATION .... 682

**EDITORIAL—**

FOOTBALL .... 683
THE SOUTHERN SURGICAL AND GYNECOLOGICAL ASSOCIATION .... 685

**SELECTIONS AND ABSTRACTS—**

OPHTHALMOLOGY AND OTOLOGY .... 688
MISCELLANEOUS .... 694

**MEDICAL ITEMS** .... 708
**BOOK REVIEWS** .... 713
**MEDICAL ITEMS—CONTINUED** .... x, xii, xiv, xvi
**ADVERTISERS' INDEX TO ADVERTISEMENTS** .... xviii

